# Does increased interdisciplinary contact among hard and social scientists help or hinder interdisciplinary research?

**DOI:** 10.1371/journal.pone.0221907

**Published:** 2019-09-04

**Authors:** Karolina Urbanska, Sylvie Huet, Serge Guimond

**Affiliations:** 1 Laboratoire de Psychologie Sociale et Cognitive, Université Clermont Auvergne, Clermont-Ferrand, France; 2 Laboratory of Engineering for Complex Systems, Irstea, Aubière, France; University of Bucharest, ROMANIA

## Abstract

Scientists across disciplines must often work together to address pressing global issues facing our societies. For interdisciplinary projects to flourish, scientists must recognise the potential contribution of other disciplines in answering key research questions. Recent research suggested that social sciences may be appreciated less than hard sciences overall. Building on the extensive evidence of ingroup bias and ethnocentrism in intergroup relations, however, one could also expect scientists, especially those belonging to high status disciplines, to play down the contributions of other disciplines to important research questions. The focus of the present research was to investigate how hard and social scientists perceive one another and the impact of interdisciplinary collaborations on these perceptions. We surveyed 280 scientists at Wave 1 and with 129 of them followed up at Wave 2 to establish how ongoing interdisciplinary collaborations underpinned perceptions of other disciplines. Based on Wave 1 data, scientists who report having interdisciplinary experiences more frequently are also more likely to recognise the intellectual contribution of other disciplines and perceive more commonalities with them. However, in line with the intergroup bias literature, group membership in the more prestigious hard sciences is related to a stronger tendency to downplay the intellectual contribution of social science disciplines compared to other hard science disciplines. This bias was not present among social scientists who produced very similar evaluation of contribution of hard and social science disciplines. Finally, using both waves of the survey, the social network comparison of discipline pairs shows that asymmetries in the evaluation of other disciplines are only present among discipline pairs that do not have any experience of collaborating with one another. These results point to the need for policies that incentivise new collaborations between hard and social scientists and foster interdisciplinary contact.

## Introduction

Complex problems faced by our society such as climate change are unlikely to be overcome by a single academic discipline [1]. Despite barriers such as lower funding rates [2] or limited institutional support [3], interdisciplinary research is growing [4] and some funders even expect research teams to be interdisciplinary. Benefits and costs of interdisciplinary endeavours are hotly discussed among scientific community but simultaneously, little is known about the processes underlying willingness to engage in interdisciplinary collaborations. For interdisciplinary research to bear fruits, scientists must take a step outside their own subject matter and recognise the intellectual contribution of other disciplines to inspire novel ways of thinking in addressing these big issues. Otherwise, seeing one’s own discipline as the only valid perspective can genuinely hinder the development of innovative interdisciplinary research. Indeed, interdisciplinary research is sometimes associated with lower productivity in terms of papers published [5]. The aim of this empirical article is to examine the experiences of scientists from a broad range of academic disciplines as they encounter and work with researchers from other disciplines. Addressing the ‘how’ of interdisciplinary research, we test whether, in line with the contact theory [6], interdisciplinary contact can foster positive perceptions of other disciplines.

### From mono- to interdisciplinary research

Throughout this paper, we refer to any research involving two or more individuals from different disciplinary perspectives with the goal of producing new knowledge as interdisciplinary research. Scientific disciplines differ between one another, each involving different subject matter and methodologies. Researchers are highly socialised within those circles as most university departments are compartmentalised into specific disciplines. One can reason about academic disciplines as cultures in the sense that physical departmental divisions foster the transmission of core values within the system and over time [7]. Indeed, research has shown that there are striking differences in attitudes, beliefs and values across disciplines [8]. Moreover, there is evidence of disciplinary socialisation effects: over the course of their training, students acquire a set of beliefs and values and learn different ways of explaining societal phenomena [8].

What happens, therefore, when a pair of disciplines that may not have a shared knowledge on a research question work together? Chiu and colleagues [7] argue that academic progression within those monodisciplinarity systems can be averse to interdisciplinary research. While viewpoint diversity may increase the quality of the output[9], Chiu et al. [7] take a rather pessimistic stance, arguing that exposure to other disciplines can prompt evaluation of differences and remarking faults in other disciplines. This can lead to conclusions that other disciplines do not perceive the problems in the same way and give rise to intellectual centrism, a form of ingroup bias whereby one displays a strong preference for own discipline over other disciplines and fails to appreciate the potential contribution of other disciplines [10]. In other words, they propose that contact with other disciplines, by harming perceptions that other scientists can contribute intellectually to the research questions of interest may hinder interdisciplinary research and collaboration.

### The ‘us’ and ‘them’ of interdisciplinary science

Seemingly consistent with Chiu et al.’s perspective [7], decades of research in carefully controlled laboratory settings have shown that the mere categorization of people into ‘us’ and ‘them’ is sufficient to elicit intergroup bias, a systematic tendency to favour the ingroup over the outgroup [11,12]. Because ingroup bias arises in minimal group settings even early in life, mere group membership is a powerful explanation for why people exhibit a strong preference for ingroups [13]. Nevertheless, the social status of the group matters for the displays of intergroup bias. In all complex societies, intergroup relations are organized as group-based hierarchy, with some groups enjoying higher power and status than others [14,15]. Meta-analytic research has revealed that for both artificial groups created in the laboratory and real-life groups, intergroup bias is stronger among members of higher status groups than among members of lower status groups [16]. Consistent with social identity theory [15], this is especially likely to occur when the status hierarchy is perceived as stable and legitimate. In such conditions, members of higher status groups simply emphasise their higher value especially in competence-related domains in order to justify their higher status [17], whereas members of low status groups tend to display no bias or even a bias in favour of the outgroup, acknowledging the status hierarchy [18].

While researchers from different disciplines may arguably be of similar social status in the eyes of the public, it is also evident that some disciplines are perceived with higher regard and status than others [8]. Common sense distinctions between “hard” and “soft” sciences suggest that some disciplines are perceived as more prototypical of the scientific ethos than others (e.g., see [19, 20]). Generally, there is an agreement that researchers coming from hard sciences such as physics or biology may more readily be perceived as ‘real’ sciences, conquering discovery of scientific laws. For social sciences such as sociology or economy, on the other hand, the study of human behaviour is highly contextualised, making it more difficult to advance and test theories in highly controlled environments. Indeed, the idea that the sciences are hierarchical is certainly not a new one and it dates back to almost 200 years [21], The question, however, is whether these hierarchies impact how scholars in those disciplines perceive one another.

Reflecting the hierarchical rhetoric, scholars have noted that methodologies used by social scientists may appear as more straightforward than those employed by hard sciences; social scientists’ expert contribution may be deemed unnecessary [22,23]. Moreover, the suggestions that “social scientists are less likely than researchers in other disciplines to want to participate in interdisciplinary projects” ([3], p. 525) and that they are “less optimistic about the challenges involved in interdisciplinary working” [24], p. 13) are not uncommon, even when there is little empirical evidence to back them up. In line with those characterisations, it may be expected that those belonging to relatively higher status hard sciences would display a bias in favour of their own academic discipline in contrast to the social sciences. To our knowledge, only one study has so far assessed whether such bias may exist. Kirby, Jaimes, Lorenz-Reaves, and Libarkin [25] have recently analysed data from some 400 earth scientists showing that they perceive social sciences as significantly less competent than the natural sciences, supporting the notion that social sciences are perceived as being of lower status. There was a silver lining, however: those who reported having some experience of working with social scientists held more positive views about the competence of social scientists compared to those with no experience. This highlights the potential importance of interdisciplinary contact and prompts the question of whether exposure to different disciplines may actually have positive effects on perceptions of other sciences. Can working with individuals from disciplines other than one’s own foster increased appreciation for these disciplines rather than simply exposing faults and differences?

### Interdisciplinary contact is an intergroup contact

A rich history of research on the effects of intergroup contact counting at least 700 studies shows that interacting with different others tends to reliably decrease intergroup bias and ethnic prejudice [26]. Any form of actual interaction between members of clearly defined groups usually counts as contact. This research tradition is known as the contact hypothesis [6]. Contact, when perceived to be positive by those who engage in it, is argued to ‘work’ because it facilitates a reduction of anxiety [27]. While most of the attention in the area has been on reducing intergroup bias, contact can also promote harmony between groups and foster diversity values [28] among other outcomes [29].

Intergroup contact can take many forms. Allport, for example, specified the ingredients that should increase the chance that high-quality contact will reduce biases. These elements are commonly referred to as conditions of contact. They state that: (a) group members should be of equal status, (b) work towards the same goal, (c) cooperate with one another, and (d) receive institutional support. Surprisingly, the extent to which these conditions are met has rarely been given attention [30]. If anything, in their meta-analysis, Pettigrew and Tropp argued that these conditions are not essential for contact to have positive effects [26]. In terms of interdisciplinary research, empirical evidence on the impact of contact is extremely limited and it has not been established whether benefits of intergroup contact can extend to increasing positive attitudes between scientists from different disciplines. Understanding which aspects of collaborations in interdisciplinary research are important in promoting more positive and more frequent contact is important in encouraging such projects. In line with this, some suggested that feeling of having input into the collaboration predicts more frequent engagement in interdisciplinary collaboration [31] and that the lack of institutional support may well be a barrier to interdisciplinary research [32]. While there is some data to suggest that more senior scientists engage in more frequent interdisciplinary collaborations [33], the empirical evidence focusing on both frequency and quality of collaborations and how they are related not only to demographics, but also to the four elements of contact, however, is lacking.

On the other hand, contact can also differ in its occurrence rates and its duration. In the context of interdisciplinary collaborations, it means that researchers can feel that their work is more or less frequently carried out with colleagues from other disciplines overall. Both the frequency and quality of contact contribute to more positive evaluations between social groups [34]. Although evaluating contact frequency and quality is a common approach within psychological science research, such evaluations tell us little about the specific impact of collaboration pairs and their dynamics. This is where the social network approach can shed more light on how the nature and the dynamics of interdisciplinary research may impact perceived intellectual contributions of other disciplines [35]. The social network approach permits the evaluation of social interactions whereby the focus falls on the relationship between specific nodes that interact with one another [36]. A node represents an individual, or a group of individuals sharing, for example, the same discipline. Moreover, these relations can exist on multiple layers with each layer characterising a different type of relationship between two nodes [37]. Therefore, scientists coming from discipline X can position themselves in relation to other disciplines by compartmentalising the type of interdisciplinary contact they have had. We propose that this can be conceptualised at four levels: (1) scientists from discipline X have no direct experience of working with someone from discipline Y, (2) scientists from discipline X have a recent new collaboration with discipline Y, (3) scientists from discipline X have an experience of working with discipline Y in the past, but not currently in the present, and (4) scientists from discipline X have a continuing collaborative relationship with discipline Y. For example, an engineer could have an ongoing collaboration with a sociologist, but maybe they have only collaborated once in the past with a psychologist. Those two types of collaboration, integrated into two layers of analysis, will have varied contributions to how the engineer perceives not only sociologists and psychologists separately but also social sciences more generally.

### The present research

The present research set out to investigate the nature and consequences of interdisciplinary contact in a two-wave online survey of researchers from a broad range of academic disciplines. Scholars need to recognise the contribution of other disciplines and perceive a sense of commonality to break down the strong ‘us’ and ‘them’ divisions in academic disciplines [13]. The first aim of the study was to examine the relationships between the properties of interdisciplinary contact (its frequency, quality, and temporal stability) and their impact on perceptions of intellectual contribution and perceived commonality with other disciplines. Given the evidence for the positive effects of contact, it was expected that having more frequent and more positive contact would be positively related to the perceived intellectual contribution of other disciplines. In addition to participant-level evaluations, we carried out a multilevel network analysis to evaluate how pairs of specific disciplines, based on the temporal stability of these collaborations, impacted perceptions of intellectual contributions of other disciplines. To evaluate the effect of collaboration history between discipline pairs, we compared disciplines that have never collaborated with one another with those for which a new collaboration between Wave 1 (W1) and Wave 2 (W2) has been reported. If commencement of a new collaboration requires an initial perception of intellectual contribution of any discipline, we expect perceived intellectual contribution at W1 to be higher among participants who have begun a new collaboration between W1 and W2 than among participants who do not report any collaboration history. Furthermore, we can evaluate whether perceptions of intellectual contribution were higher among those with sustained collaborations reported at both W1 and W2 than only those who recently began a collaboration. To provide further evidence on what may encourage interdisciplinary contact, we explored whether conditions of contact outlined by Allport [6] predicted how frequently researchers engage in interdisciplinary contact and whether these interactions were generally positive or negative. Our prediction was that all four conditions (equal status between group members, a common goal, warm cooperation, institutional support) would predict more frequent and more positive interdisciplinary contact.

The second aim of the study related to evaluating the impact of scientific hierarchies between hard and social sciences on the interdisciplinary collaborations and perceptions of other disciplines. Following Kirby et al. [25], it was expected that social sciences disciplines will be perceived as less capable to contribute intellectually to scientific research questions than hard science disciplines. Moreover, going beyond Kirby et al. [25] and following the literature on the role of status in intergroup bias, it was hypothesised that there would be an asymmetry in the way scientists perceive other disciplines as a function of their group membership. More specifically, we predicted hard sciences, as a higher status discipline, would display a stronger ingroup bias, that is, a stronger preference towards other hard science disciplines than social science disciplines.

We build on the existing research in multiple ways. First, extending on Kirby et al.’s research, we sample researchers at different career stages and from multiple scientific disciplines. Second, we provide the first direct test of the effect of interdisciplinary contact in terms of its quantity and quality on the scientists’ perceptions of other disciplines. Third, we take a dynamic approach in evaluating the nature of these collaborations across time, how they relate to perceptions of intellectual contribution of other disciplines and perceived commonality, and assessing the influence of beginning a new research collaboration (versus continuing an existing one or not having any direct collaborative experience with certain disciplines). Fourth, we provide some initial evidence on the specific aspects of interdisciplinary collaborations which contribute to more frequent and more positive interdisciplinary contact. Overall, this research strived to provide essential empirical evidence that can stimulate discussion regarding the processes underpinning interdisciplinary research and how to best promote future interdisciplinary collaborations.

## Materials and methods

This two-wave study was conducted as a part of a larger research programme which aimed to promote interdisciplinary approaches bridging social and hard sciences to build more adaptive and resilient societies. The research programme consisted of 12 partner universities in seven countries, representing 12 academic disciplines of the research programme partners (see Table 1). We sent invitation emails to all research partners to complete an online survey and asked them to forward the link to their colleagues and collaborators. The research was presented as a study of “the underpinnings of innovative research, including the mechanisms of interdisciplinary research”. In total, 160 academic researchers from the hard sciences and 120 researchers from social sciences participated in the online survey at W1. They reflected all levels of seniority with 26 PhD students, 19 postdoctoral researchers, 53 assistant professors, 104 associate professors, 37 full professors and 37 head of departments (information missing for 4 participants). They were affiliated with 26 different countries. Following the completion of W1, 220 out of 280 participants left their email addresses to be contacted of which 59% participated (*n* = 129). Attrition analysis suggests that the dropout rates are more systematic in terms of demographics with those who are in more senior positions and hard sciences more likely to drop out at W2. However, there were no differences in the key measured variables between those who completed both waves and those who dropped out (see supplementary analyses for details: https://osf.io/6spxh/). W1 took place between December 2017 and April 2018 whereas W2 between November and December 2018. Both parts of the study were available online and took around 10 minutes to complete.

**Table 1:** Rotated (Oblimin) factor loadings of intellectual contribution and commonality items on two factors (social and hard sciences) extracted with principal axis functioning. Number of participants representing each discipline is also displayed.

			Intellectual contribution		Commonality	
Classification	Discipline	W1 *n*	Factor 1	Factor 2	Factor 1	Factor 2
Social science	Cognitive science	9	0.73		0.70	
	Comp SS	23	0.81		0.77	
	Economy	10	0.71		0.61	
	Geography	11	0.62		0.41	
	Linguistics	8	0.69		0.62	
	Philosophy	2	0.70		0.65	
	Psychology	22	0.84		0.84	
	Sociology	9	0.83		0.77	
	Other (hand coded)	23				
Hard science	Computer science	28		0.66		0.71
	Engineering	18		0.70		0.76
	Mathematics	14		0.78		0.83
	Physics	41		0.77		0.79
	Other (hand coded)	61				
Missing		1				
**Total**		**280**				

Factor loadings for values below 0.40 are suppressed. Comp SS = computational social science.

Research was conducted according to the principles expressed in the Declaration of Helsinki. Participants gave written consent to participate in the study voluntarily. Their email addresses were kept confidentially and were destroyed following the W2 of the study. This research was non-interventional and without the use of deception and therefore was not required to go through the ethics committee.

Measures employed across both waves of the online survey were almost identical with an exception of demographic variables collected only at W1 and additional variables measuring conditions of contact at W2. Not all measures collected are presented here, but we list all study materials, including a list of variables not reported in this manuscript in the supplementary materials.

### Demographic variables (W1)

Information regarding gender, academic position, and country of residence was collected. These are included in the analyses for exploratory purposes. Participants also indicated their main academic discipline from a list of 12 which were classified as either hard or social science and all disagreements were discussed by all authors. Inter-rater reliability for coding of disciplines marked as ‘other’ was 87%. Factor analysis further confirmed this classification (see Table 1).

### Interdisciplinary contact measures

Participants were asked to report on the interdisciplinary collaborations they had in terms of their frequency and quality. The single‐item measure for each dimension was adapted from Tam and colleagues’ research [38]. In W1, they were asked about their contact experiences in their career up to date and at W2, they reported on more recent collaborations (since January 2018). For contact frequency, they stated how frequently they work with others who come from another discipline (1 = *neve*r; 7 = *very frequently*). For contact quality, they rated how positive or negative these interdisciplinary experiences were overall (1 = *extremely negative*; 7 = *extremely positive*).

**Fig 1:**
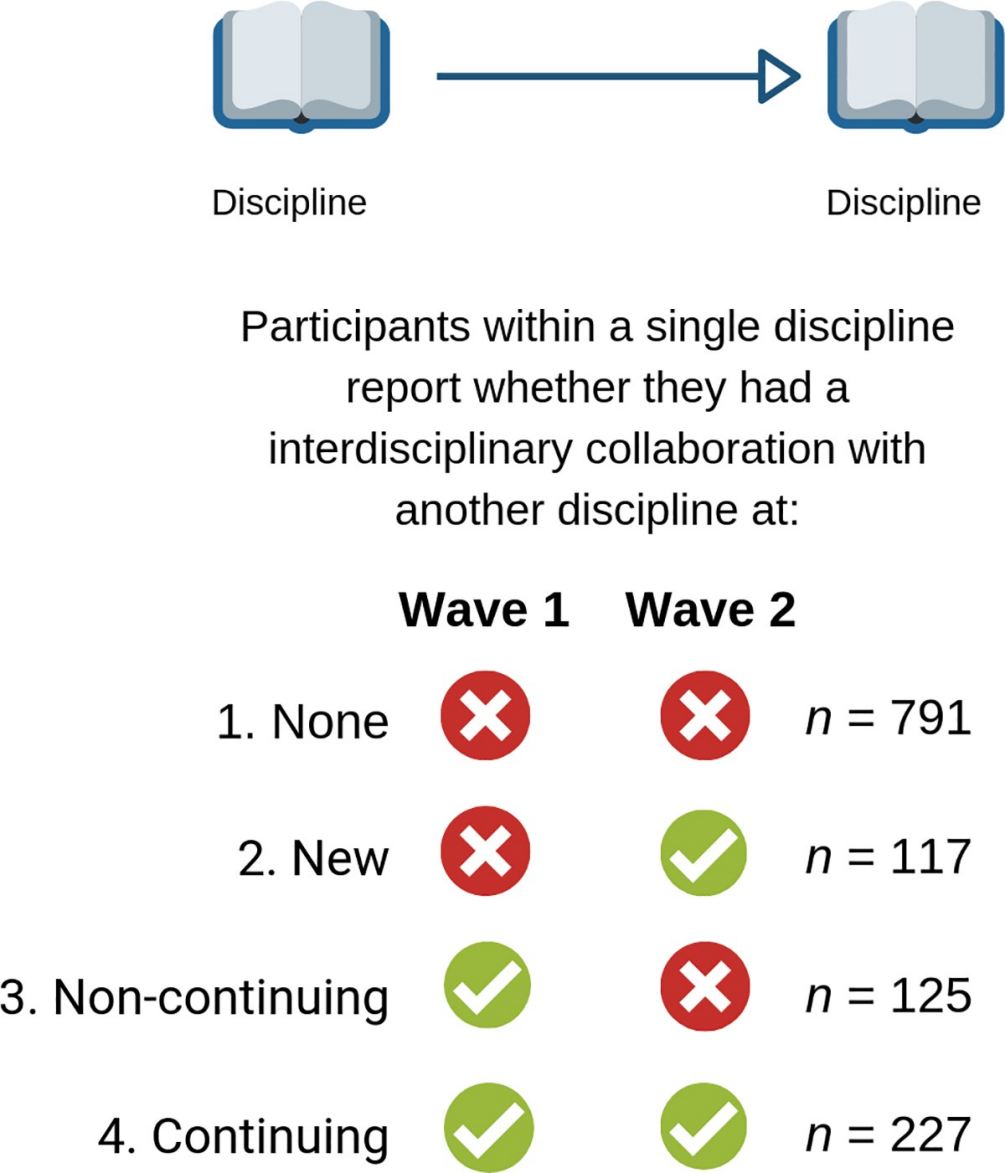
Categorisation of types of collaboration pairs within networks based on reporting an interdisciplinary collaboration at W1 (in their career up to date) or at W2 (in between the two waves). Number of discipline pairs included in the analysis is provided on the right-hand side.

Moving on to questions regarding specific disciplines, participants first specified which disciplines they collaborated in the past (W1) and with which disciplines they collaborated since January 2018 (W2). For those who completed both waves of the study, information regarding all pairs of discipline was extracted, creating 11 scores for each participant. Four categories of histories of collaboration pairs were derived from this information: (1) no collaboration reported in either waves, (2) a new collaboration pair reported in W2, (3) a collaboration pair that was reported in W1, but was not continued, and (4) a continuing collaboration pair reported in both waves (see Fig 1).

### Outcome measures: Intellectual contribution and perceived commonality

We estimated the extent to which researchers viewed other disciplines as capable of contributing to intellectual knowledge underpinning the relevant research questions. All 12 disciplines were displayed and for each, participants were asked about the extent to which other listed disciplines can contribute to the research questions the participant studies (1 = *not at all*; 7 = *a great deal*). There was also an option to indicate that one of the listed disciplines is one’s own in which case the item has been coded as missing and excluded from the scale construction. A higher score reflected a higher perception of intellectual contribution of scientific disciplines other than one’s own (α_W1_ = 0.79; α_W2_ = 0.78). For this measure, we further split the scale into perceived intellectual contribution related to hard science disciplines and social science disciplines separately based on Table 1. Following this exercise, the internal reliability remained high for the contribution of hard sciences (α_W1_ = 0.70, α_W2_ = 0.75) and social sciences (α_W1_ = 0.87, α_W2_ = 0.83).

Perceived commonality between one’s own discipline and other disciplines was assessed using Venn diagrams. Participants had to choose one out of five degrees of overlap ranging from no overlap at all (1) to almost a total overlap (5). This measure was collected at both wave points. The ratings for each discipline (other than participants’ own) were merged into one score with a higher score reflecting a higher degree of perceived commonality with other disciplines (α = 0.73 and 0.71 in W1 and 2 respectively). As with the intellectual contribution items, we further derived two variables for hard and social science targets. These also had a high internal reliability (hard science α_W1 and 2_ = 0.75; social science: α_W1_ = 0.81; α_W2_ = 0.83). Providing evidence for the validity of these outcome measures, the perceived commonality measure was strongly correlated with the perceived intellectual contribution, *r*(228) = 0.65, *p* < 0.001 and *r*(119) = 0.73, *p* <0.001 in W1 and W2, respectively.

### Conditions for contact

Finally, W2 further included items assessing Allport’s conditions for positive contact. Since no existing measure was found, new items were created. On a scale from 1 (*not at all*) to 7 (*completely*), participants were asked about the extent to which both sides of collaboration were working to achieve the same goal (*goals*), the collaboration was supported by their department or lab (*institution*), the interdisciplinary collaboration was meaningful and warm (*warm*). To assess whether collaboration was equal, one item asked about the extent to which the collaboration was between equal partners and another item, which was reverse-coded, about the extent to which some partners were dominant over others. These two items correlated highly, *r*(121) = 0.60, *p* < 0.001, and were merged into a single variable (*col_equal*).

## Open science note

All materials, data and analyses are available via the Open Science Framework project page: https://osf.io/ynerz/.

## Results

### Social or hard science?

To confirm the classification of disciplines into social and hard sciences, confirmatory factor analysis was conducted with principal axis functioning and Oblimin rotation. As the perceived intellectual contribution and perceived commonality items used the same disciplines, we used these items to confirm that in both measures, there is a clear distinction between social and hard science disciplines and thus two factors were extracted. Inspection of factor loadings suggest that there were no cross loadings and all disciplines consistently loaded on the relevant factors across two measures as it was originally classified by the authors (see Table 1).

### The impact of interdisciplinary contact on perceived intellectual contribution and commonality

**Fig 2:**
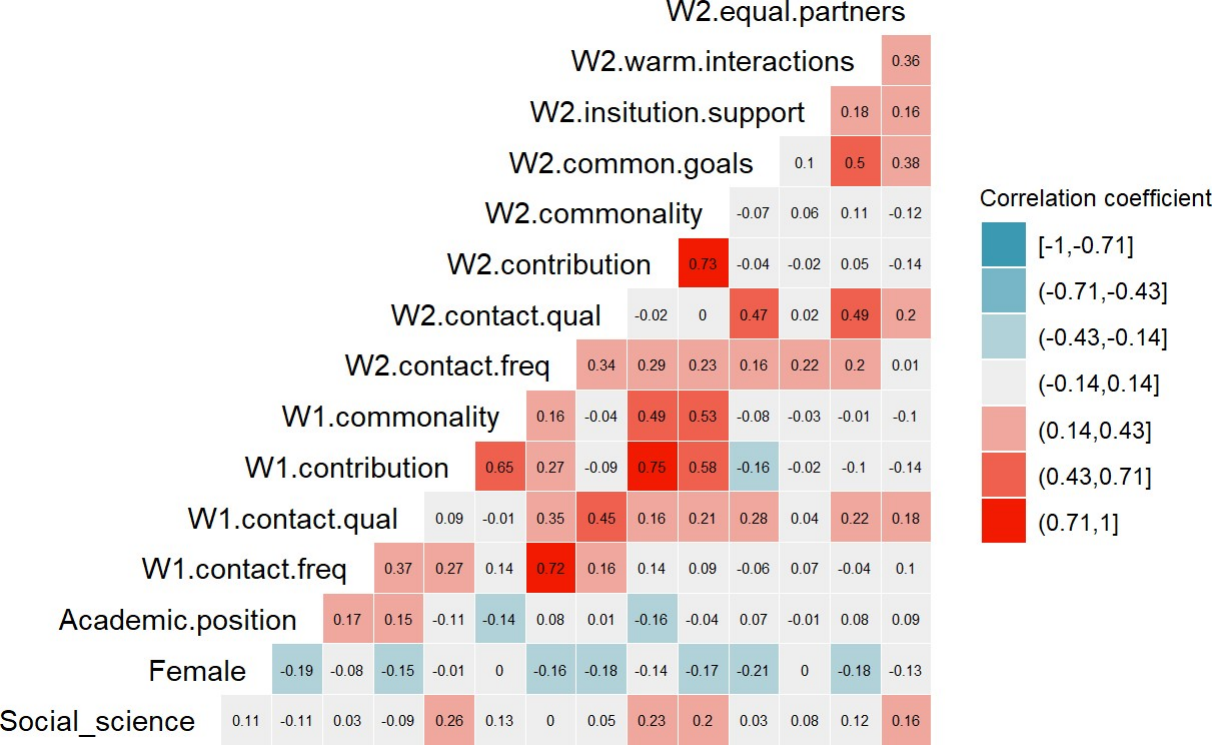
Cross-correlation heat map with correlation coefficients among demographical information and key variables measured in W1 and 2. Relevant correlations are colour-coded based on the strength of the correlation (and not based on the null hypothesis significance test).

To investigate the relationship between interdisciplinary contact and the outcome variables, we first inspected cross-correlations between those variables at both waves. Fig 2 shows that higher frequency of reported interdisciplinary contact was related to a higher perceived intellectual contribution of other disciplines at W1, *r*(241) = 0.27, *p* < 0.001. Moreover, this pattern of results was further replicated at W2, *r*(128) = 0.29, *p* < 0.001. Higher frequency of interdisciplinary contact was also related to higher perceived commonality, albeit the effects were weaker: *r*(236) = 0.14, *p* = 0.037 for W1 measures and *r*(121) = 0.23, *p* = 0.012 for W2 measures. Therefore, there is initial evidence that more frequent interdisciplinary contact is related to more positive perceptions of other disciplines. Contact quality, however, was not related to perceived intellectual contribution for W1 measures, *r*(237) = 0.09, *p* = 0.152 nor W2 measures, *r*(120) = -0.02, *p* = 0.831; nor it was related to perceived commonality for W1 measures, *r*(233) = -0.01, *p* = 0.925, and W2 measures, *r*(104) = -0.04, *p* = 0.716.

To control for demographic factors, additional variables were also entered into a multiple linear regression predicting intellectual contribution and commonality at W1, including hard/soft science discipline, gender, and academic position. This model explained 14% of variance, *F*(5, 230) = 8.37, *p* <0.001. Contact frequency remained the strongest predictor of perceived intellectual contribution, β = 0.29, *t* = 4.70, *p* < 0.001. While gender was a non-significant predictor, β = -0.04, *t* = -0.63, *p* = 0.528, being a hard scientist (versus social scientist), β = -0.25, *t* = -4.06, *p* < 0.001 and having a less senior academic position, β = -0.13, *t* = 2.12, *p* = 0.035 were both associated with higher perceptions of intellectual contribution of other disciplines. For perceived commonality, the predicting variables were of similar effect, but slightly weaker, explaining only 7% of the variance, *F*(5, 226) = 4.28, p <0.001, with the less senior position, again, associated with higher perceived commonality with other disciplines (see Table 2). Therefore, reporting more frequent (but not necessarily more positive) interdisciplinary contact overall was related to more positive perceptions of other disciplines.

**Table 2:** The effects of interdisciplinary contact on the perceived intellectual contribution of other disciplines and perceived commonality with them.

Predictor	Intellectual contribution		Commonality	
	β	t	β	t
Social science^a^	0.25	4.09***	0.10	1.67
Female^b^	-0.03	-0.41	-0.05	-0.70
Academic position	-0.13	-2.08*	-0.16	-2.44**
Contact frequency	0.25	3.79***	0.24	3.48***
Contact quality	0.05	0.82	-0.08	-1.16
**Adjusted R**^**2**^	0.14		0.07	

* *p* < 0.05

** *p* <0.01

*** *p* < 0.001.

^a^57% hard science (coded 1), 43% social science (coded 2).

^b^70% male (coded 1), 30% female (coded 2).

### Elements of positive contact

Finally, as a part of exploratory analyses in W2, four conditions of positive contact as outlined by Allport [6] were examined in the context of reported contact quality and contact frequency (see Table 3). In the regression model predicting contact frequency model, only a small effect of institutional support was observed: those who perceived their institution to be more supportive of interdisciplinary collaborations were more frequently engaging in them, β = 0.20, *t* = 2.20, *p* = 0.030. For the contact quality, the model suggests that higher quality contact was associated with a higher perception that both sides of collaborations shared a common goal, β = .31, *t* = 3.39, *p* <0.001, and the perception that the nature of collaboration was warm, β = 0.36, *t* = 3.97, *p* <0.001. Institutional support and equal-level partnership were non-significant predictors. Both models were statistically significant, *F*(4, 117) = 2.92, *p* = 0.024, Adjusted *R*^2^ = 0.06 and *F*(4, 117) = 13.47, *p* <0.001, Adjusted *R*^2^ = 0.29 for contact frequency and contact quality, respectively. In neither of the models, perception that the collaboration was between equal partners predicted contact frequency or contact quality.

**Table 3:** Regression model with conditions of contact.

	Contact frequency		Contact quality	
	β	t	β	t
Goals	0.10	0.95	0.31	3.39***
Institution	0.20	2.20*	-0.08	-0.98
Warm	-0.16	1.53	0.36	3.97***
Col_equal	-0.12	-1.23	-0.04	-0.43
**Adjusted R**^**2**^	0.06		0.29	

* p < 0.05

*** p < 0.001.

### Perceptions of intellectual contribution and commonality with other disciplines across hard and social science group lines

For exploratory purposes, we first compared those in hard and social science disciplines on the number of key outcomes to evaluate any differences using a series of independent t-tests. On almost all measures, there were no significant differences between participants from hard and social sciences. The results show that while hard scientists do not report engaging in a more frequent or a more positive interdisciplinary contact than social scientists either in W1 or W2, they did report perceiving other disciplines as contributing less to the research questions they are studying than the social scientist participants (see Table 4 for statistics).

**Table 4:** Differences between hard scientists and social scientists on the key measures.

	Hard science		Social science			
	*M* (*SD*)	*n*	*M* (*SD*)	*n*	*t*	*d*
**Contact frequency**						
W1	5.63 (1.54)	160	5.72 (1.59)	119	-0.48	0.06
W2	5.22 (1.72)	63	5.24 (1.89)	63	-0.05	0.01
**Contact quality**						
W1	5.91 (1.06)	158	5.71 (1.03)	117	1.53	0.19
W2	5.77 (1.01)	60	5.86 (1.02)	59	-0.52	0.10
**Contribution**						
W1	3.48 (1.13)	139	4.10 (1.10)	103	-4.23***	0.55
W2	3.41 (1.15)	62	3.91 (0.93)	63	-2.66**	0.48
**Commonality**						
W1	2.49 (0.68)	133	2.66 (0.60)	104	-1.93	0.25
W2	2.50 (0.71)	58	2.78 (0.62)	58	-2.23*	0.41

* *p* < 0.05

** *p* < 0.01

*** *p* < 0.001.

**Fig 3:**
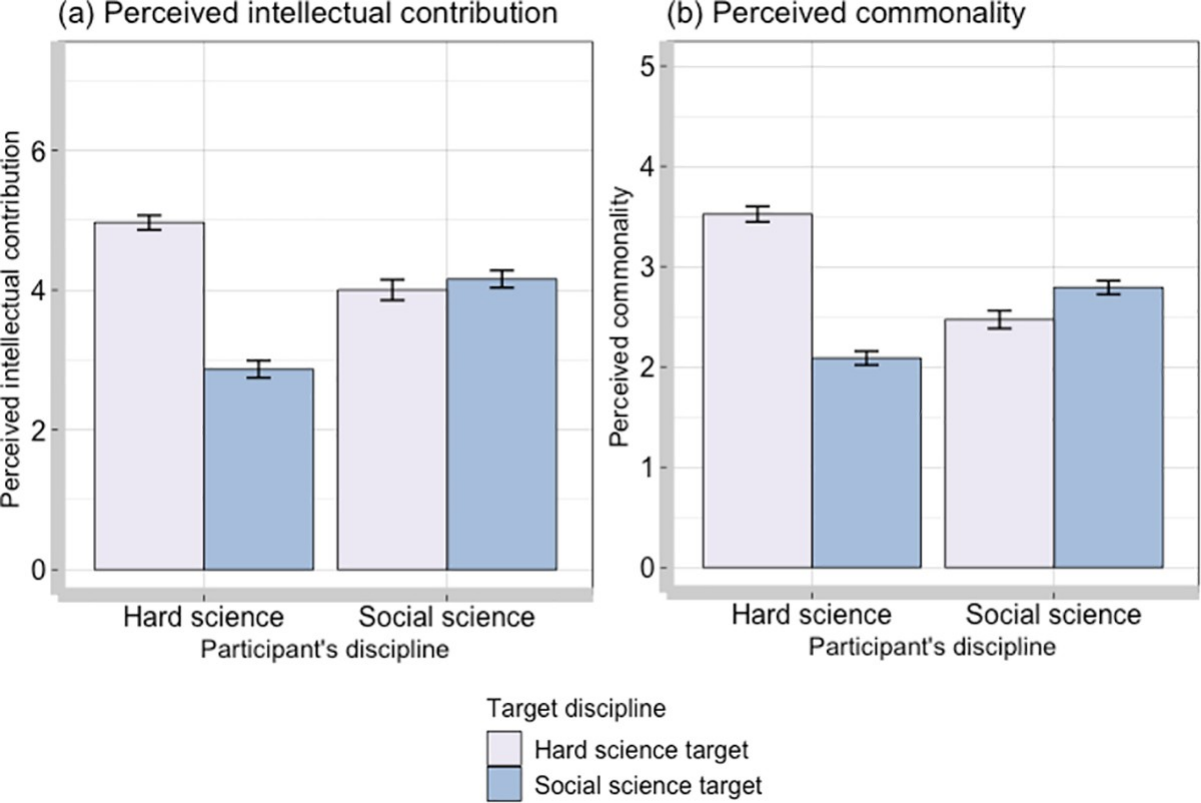
**(a) Perceived intellectual contribution (measured on a scale 1–7) and (b) perceived commonality (measured on a scale 1–5) in relation to other hard science and social science disciplines as a function of participant’s own disciplinary belonging.** Error bars represent standard error.

Next, we tested whether social science disciplines were perceived as more able to contribute intellectually than hard science disciplines in the eyes of hard scientist participants and social science participants and vice-versa. To evaluate this, a 2 (Participant’s discipline: Hard science versus Social science; between factor) x 2 (Target discipline: Hard science versus Social science; within factor) mixed ANOVA with the perceived intellectual contribution as an outcome was conducted (see Fig 3A). Scientists from hard and social sciences did not differ in their overall levels of perceived intellectual contribution, *F*(1,85) = 0.13, *p* = 0.716, η^2^_G_ < 0.01. However, there was a medium main effect of target discipline on intellectual contribution, *F*(1,85) = 20.08, *p* < 0.001, η^2^_G_ = 0.07. Participants generally rated the intellectual contribution of hard science disciplines as higher (*M* = 4.55, *SD* = 1.43) than social science disciplines (*M* = 3.41, *SD* = 1.51).

Moreover, there was a significant Participant Discipline x Target Discipline interaction on intellectual contribution, *F*(1,85) = 14.41, *p* < 0.001, η^2^_G_ = 0.05. To break down this interaction, two paired t-tests were carried out with a Bonferroni alpha correction applied (p = 0.0025). For those coming from hard science backgrounds, participants viewed hard science disciplines as being able to contribute intellectually significantly more (*M* = 4.96, *SD* = 1.23) than social science disciplines (*M* = 2.87, *SD* = 1.45), t(139) = 13.74, p <0.001. This effect was large, *d* = 1.16. For those coming from social science disciplines, there was no difference in their perceptions of intellectual contribution from hard or social science disciplines, *t*(103) = -0.97, *p* = 0.337, d = 0.09. The analysis shows a large asymmetry in the way hard scientists and social scientists perceive their potential intellectual contributions.

Next, we proceeded to test for Participant Discipline x Target Discipline interaction on perceived commonality. There was a small main effect of participant’s discipline this time with hard science participants perceiving overall more commonality with other disciplines (*M* = 2.81, *SD* = 1.12) than social science participants (*M* = 2.63, *SD* = .84), *F*(1, 244) = 4.27, *p* = 0.04, η^2^_G_ = 0.01. Furthermore, the main effect of target discipline further replicated the previous analyses with hard science disciplines perceived as having more in common with (*M* = 3.06, *SD* = 1.06) than with social science disciplines (*M* = 2.39, *SD* = .84), *F*(1, 244) = 64.60, *p* < 0.001, η^2^_G_ = 0.10.

However, of particular interest in the present research was the interaction effect. The Participant Discipline x Target Discipline interaction produced a large effect on perceived commonality, *F*(1, 244) = 159.93, *p* <0.001, η^2^_G_ = 0.21 (see Fig 3B). As with previous analyses, we analysed this interaction using two paired t-tests with a Bonferroni alpha correction applied (*p* = 0.0025). Among those from hard science background, they perceived more commonality with other hard science disciplines (*M* = 3.52, *SD* = .91) than with social science disciplines (*M* = 2.08, *SD* = .81), *t*(138) = 15.57, *p* < 0.001, *d* = 1.32. For social science participants, they perceived more commonality with social science disciplines (*M* = 2.79, *SD* = 0.71) than hard science disciplines (*M* = 2.47, *SD* = 0.92), but this difference was not statistically significant under the adjusted alpha, *t*(106) = 3.10, *p* = 0.003, *d* = 0.30. This analysis further confirms that there are asymmetries in the way hard scientists and social scientists perceive one another.

### Does contact aids positive perceptions or do positive perceptions enable contact?

Finally, we present results from the network analysis of 1,260 discipline pairs based on the history of collaboration (see Fig 1). Given the complexities of these analyses, we only report the perceived intellectual contribution as the outcome. Analyses regarding the perceived commonality are available in the supplementary materials (https://osf.io/6spxh/). Using this approach, we evaluated whether the history of collaboration directly affects perceptions of intellectual contribution. To this end, we conducted a one-way ANOVA with the collaboration history (none, new, non-continuing, and continuing) as the factor predicting perceived intellectual contribution.

**Fig 4:**
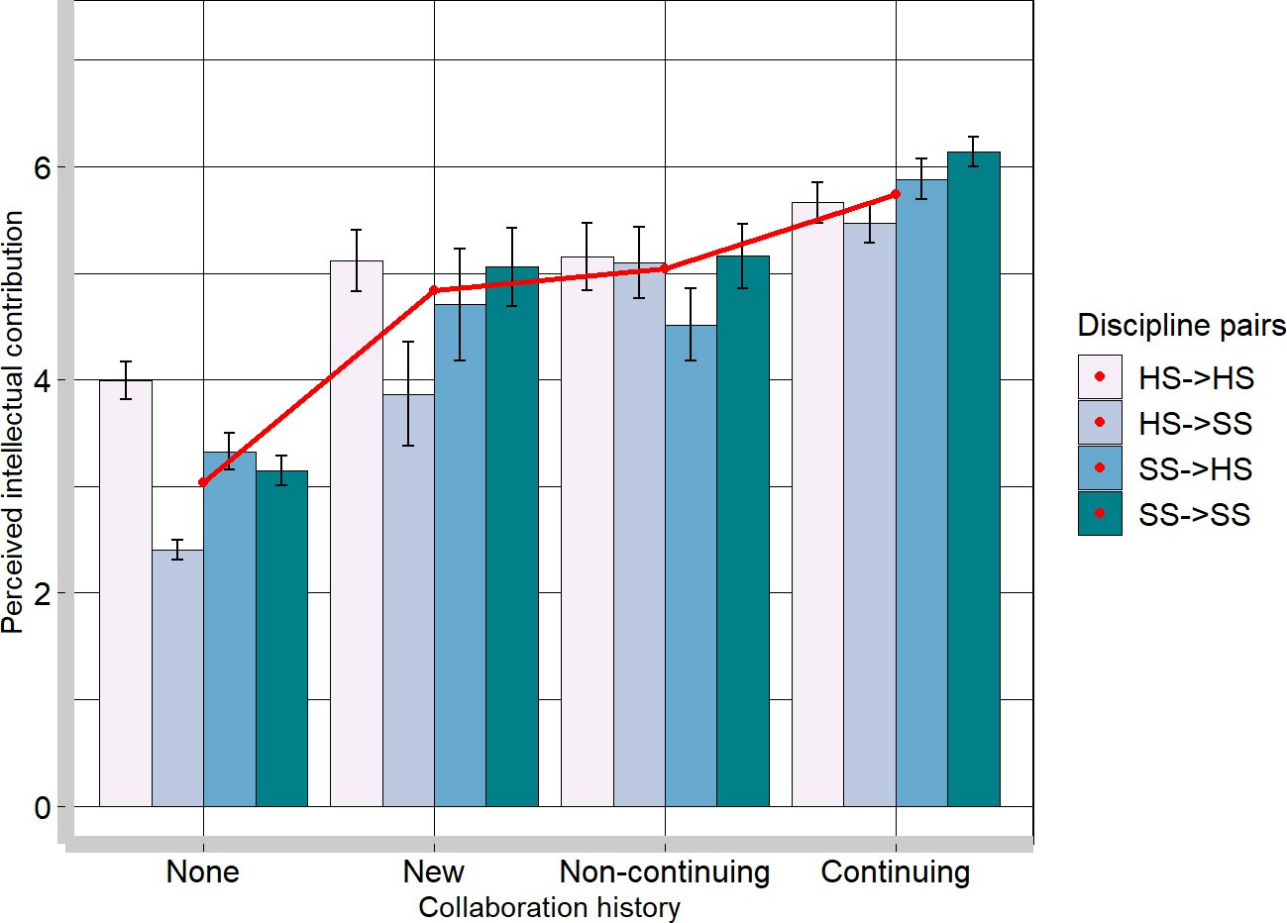
Perceived intellectual contribution of discipline pairs as a function of types of collaboration history. **In the discipline pairs, “HS->SS” means that a hard science participant evaluated social science discipline targets**. Red line represents aggregated mean score for the collaboration history type. Error bar represents standard error. HS = hard science; SS = social science.

The size of the main effect of collaboration type on perceived intellectual contribution was medium-to-large, *F*(3, 1365) = 187.10, *p* <0.001, *f* = 0.64 (see Fig 4). A comparison of four types of collaboration histories using Tukey’s test showed that among non-collaborating discipline pairs, perceived intellectual contribution of other disciplines (*M* = 3.03, *SD* = 1.90) was considerably lower than among newly established disciplinary pairs (*M* = 4.84, *SD* = 1.78) 95% CI [1.32, 2.29] as well as in comparison to those with non-continuing collaborations (*M* = 5.04, *SD* = 1.70) 95% CI [1.59, 2.43]. The difference between new and non-continuing collaborations was non-significant, 95% CI [-0.40, 0.81]. This suggests that a recognition of intellectual contribution may be initially necessary to enable the future interdisciplinary collaboration: discipline pairs that launched a new interdisciplinary collaboration within the coming months had a higher appreciation of the intellectual contribution of those disciplines months before the collaboration initiation.

Furthermore, discipline pairs that reported a continued collaboration had a significantly higher level of perceived intellectual contribution (*M* = 5.74, *SD* = 1.34) than new discipline pairs, 95% CI [0.36, 1.44] and non-continuing pairs, 95% CI [0.21, 1.18]. Therefore, there is evidence that having a sustained, pro-longed collaboration can further augment the perceived intellectual contribution of other disciplines.

### Does contact diminish the asymmetries?

Using the data of discipline pairs, we also investigated whether previously reported asymmetries in the way that hard and social sciences perceive one another may change as a result of collaboration history. To this end, we carried out four one-way ANOVAs, each representing a type of collaboration history (none, new, non-continuing, continuing) with discipline pair (hard scientists evaluating other hard science disciplines or social science disciplines or social scientists evaluating hard science disciplines or other social science disciplines). As with the previous analysis, we computed the analyses with the perceived intellectual contribution as the outcome (see Fig 4).

Among those who never collaborated, there was a significant effect of collaboration pair on perceptions of intellectual contribution, *F*(3, 721) = 25.03, *p* <0.001, *f* = 0.64. A comparison of four types of discipline pairs using Tukey’s test revealed a pattern of findings similar to the one presented in Fig 3A. Social scientists perceived other social science disciplines and hard science disciplines with whom they have never collaborated as not significantly different in their ability to contribute intellectually to relevant research questions. Hard scientists, on the other hand, for disciplines with whom they did not collaborate, perceived other hard science disciplines as being able to contribute intellectually more than other social science disciplines. For the new collaborations, *F*(3, 79) = 1.85, *p* = 0.146, *f* = 0.26, the non-continuing collaborations, *F*(3, 113) = 0.91, *p* = 0.439, *f* = 0.16, and the continuing collaborations, *F*(3, 211) = 2.64, *p* = 0.050, *f* = 0.19, the perceived intellectual contribution within the disciplinary pairs was non-significant. This demonstrates that the asymmetries in the way hard scientists and social scientists perceive one another’s contribution is likely to stem from a lack of experience working with those disciplines.

## Discussion

We tested the role of interdisciplinary contact on perceptions of other sciences with an aim to understand how intellectual centrism, a strong preference for own discipline and failure to appreciate the potential contribution of other disciplines, may be reduced. Multiple regression analyses demonstrated that those who engage in a more frequent interdisciplinary contact tend to report higher appreciation of the other disciplines’ intellectual contribution. More frequent contact was also related to an increased perception of commonality with other disciplines. These effects persisted even when controlling for demographic factors such as gender, seniority, and discipline belonging. Examination of the evolution of collaboration between pairs of discipline between W1 and W2 further showed that initial appreciation of intellectual contribution of other disciplines may be necessary to commence a new interdisciplinary collaboration in the future. However, those with a prolonged history of collaboration with the same discipline held the most positive perceptions of intellectual contribution of those disciplines. These findings are in line with the contact theory ([6], and in contrast to [7]): those who engage in more contact with social groups that are not considered their own, in this case, scientists from other academic disciplines, tend to report more favourable perceptions of those disciplines. The mere frequency of contact as opposed to its quality played a particularly important role. Scientists engaging in a more frequent collaborative endeavours with other disciplines, via exposure and getting to know these disciplines, report higher appreciation of their intellectual input. In our sample, increased interdisciplinary contact also reliably predicted increased sense of commonality with other disciplines. Moreover, continuous and sustained interdisciplinary collaborations were further elevating perceived intellectual contribution and perceptions of commonality between researchers. Therefore, increased contact between scientists of different disciplines should be actively encouraged. This could be achieved by, for example, introducing departmental policies fostering and supporting interdisciplinary endeavours. Having said that, scientists embarking on a new interdisciplinary journey have generally more positive attitudes towards disciplines with which they will start working, highlighting that a degree of intellectual recognition may be what scientists need before they can engage in interdisciplinary contact. It is possible, however, that these positive attitudes may stem from indirect contact, in other words, hearing from other colleagues about their successful interdisciplinary collaborations with another discipline (see [39]).

A multiple regression analysis also showed that perceiving local institutions as supportive of interdisciplinary collaborations was related to more frequent interdisciplinary research. This effect was small and based on a smaller W2 sample, but it is in line with previous research highlighting how institutional policies directly impact behaviour by changing norms [40,41]. Green and colleagues also recently showed that introduction of pro-multiculturalism policies is associated with more frequent and more effective contact between two social groups [42]. Based on these findings, a new hypothesis can be generated: that when scientists perceive institutional support encouraging interdisciplinary collaborations, this will increase their involvement in interdisciplinary collaborations and improve their perceptions of the potential scientific contributions of other disciplines. In terms of other conditions of contact, the analysis suggests that having common goals and warm interactions during the collaboration predicted more positive experience of interdisciplinary collaborations. Contrary to our expectations, Allport’s fourth condition, having equal status, was not related to either contact frequency or contact quality. While we cannot conclude that this condition is irrelevant in all contexts, we find no evidence that researchers need to perceive their interdisciplinary collaborators as having an equal voice or status for the collaboration to take place and be positive. One possibility is that status differences are simply not a barrier: scientific teams are often made up of members varying in seniority and expertise [43]. Addressing whether and how these conditions underlie interdisciplinary collaborations is a fruitful avenue for future research, addressing the gap in the contact research theory as advocated by Paluck and colleagues [30]. It is worth reiterating that in the present research we provided a first attempt at measuring contact conditions with items of unknown validity. More work is needed to build on this exploratory research and develop stronger measures before we can engage in confirmatory research.

### Asymmetries in perceived intellectual contribution

Our findings highlight a strong asymmetry in perceptions of other disciplines among those working in hard science disciplines and those working in social science disciplines. In line with Kirby et al. [25], the analysis demonstrated that social sciences are generally perceived as less capable of contributing to relevant research questions. Moreover, our study is the first to show that social scientists do not have different perceptions of other disciplines, regardless of whether they were reporting about hard sciences or social sciences. In other words, social scientists may not necessarily perceive other social sciences, excluding one’s own, as more able to contribute to the relevant research than hard science disciplines. The story is quite different among respondents from hard science disciplines. Hard science participants displayed a strong preference for other hard science disciplines in terms of both how much they think they contribute intellectually and how much in common they perceive to have with them. These findings resonate with observations within social psychological research demonstrating that intergroup bias is stronger among members of higher status groups than among members of lower status groups [14,16]. The present study further demonstrates this idea in a previously untested context. Moreover, the asymmetry in the way hard and social scientists perceive other disciplines is particularly strong within pairs that never had any collaborative experience. We can only speculate about the causes of those effects but the direction of the relationship is again very clear: negative perceptions of other discipline are related to a lack of contact with them. While some argue that social scientists may be perceived as partners that are unwilling [3,24] or less skilled [25] in their interdisciplinary research, the future research should consider how the visibility of social sciences could be increased to create space for interdisciplinary endeavours. One should keep in mind that these results are based on perceptions only and not whether other disciplines can actually contribute meaningfully to the relevant research question. However, if scientists perceive certain disciplines as incapable of contributing to their own research questions, this may indeed be a real first barrier to embarking on such interdisciplinary journeys.

Examining perceived intellectual contribution at discipline pair level, the asymmetry in perceptions of hard and social sciences’ contribution was smaller for those who have ongoing collaborations with different disciplines than for those who did not have any experiences of collaboration. While these asymmetries exist among those without any experience of collaboration with hard scientists displaying a strong preference for other hard science disciplines, this was no longer the case when hard scientists have experience of working with social sciences. This is in line with the findings from Kirby et al. [25] according to which those with experience of working with social scientists held more positive views about the competence of social scientists compared to those with no experience. Through the experience of collaboration, group differences in appreciation of different disciplines are considerably weakened. This again highlights the importance of encountering and working with other researchers as a pathway to growing intellectual appreciation of one another.

Worth reiterating is also that we did not set out to test the following hypothesis directly, the data suggests that those more senior in their positions tend to appreciate other disciplines less in terms of the perceived contribution and perceived commonality. This effect size was small and therefore more evidence is needed, but it is in line with previous research showing socialisation effects [8] whereby people grow to appreciate the values of their own discipline as they progress through career ranks. Another explanation could be that due to the growing appreciation of interdisciplinary research [4], early career researchers are more likely to be encouraged to pursue projects in collaboration with other disciplines. Having said that, in line with previous research [33] our data also suggests that those holding positions that are more senior reported having interdisciplinary collaborations more frequently than those earlier in their career. For this reason, future research should directly test whether monodisciplinary socialisation across career stages and within hard and social sciences strengthens ingroup biases and may be associated with more resistance to interdisciplinary research.

#### Limitations

Limitations to the present research should be acknowledged. First, not all academic disciplines were included representatively in this study and the sample was admittedly constrained by the requirements of the larger project. Social and hard sciences were also hand-coded by the authors and one may contest whether these disciplines belong to purely social versus hard sciences. We concede that this binary classification is not ideal and perhaps future research should consider the extent to which scholars feel that they strictly belong to those scientific categories. For this reason, one needs to be careful when generalising these findings to all hard science and social science disciplines. Second, while we provided participants with the definition of interdisciplinary research, it is quite likely that participants could have referred to very much neighbouring disciplines as interdisciplinary. For example, within a psychology department, a social psychologist can collaborate with a cognitive neuroscientist and well count it as interdisciplinary since it requires integration of methods or theories. In line with our data and considering the history of collaboration pairs within the disciplines we studied, interdisciplinarity may be a relatively rare phenomenon overall and even more so across hard/social science group boundaries. Relatedly, what consists of a collaboration can vary in intensity from a simple consultation to developing grant proposals together and we did not consider these complexities in the present research. Third, our findings regarding the contact conditions on contact quality and frequency were exploratory and carried out on a smaller sample in W2. Likewise, reduced sample size did not permit us to have enough statistical power to carry out relevant longitudinal tests of the reported effects.

Finally, there is no evidence that having a more favourable perception of another discipline can result in a successful interdisciplinary collaboration. This has not been directly tested in the present research. The question of what consists of “successful” interdisciplinary collaboration in itself can be extensively debated: having a collaborator with whom one enjoys working, does it consist of multiple publications, high impact factor publications, high societal impact, receiving scientific awards from peers? While this is something future research can consider, we argue that being open to such collaborations to happen when opportunities arise and frequently participating in interdisciplinary endeavours can increase the likelihood that such research will be considered fruitful and even groundbreaking. However, it is a limitation that we did not measure any indices of perceived productivity within those interdisciplinary projects such as publication success [44]. Given that our study only followed the scholars for less than a year, it was not sufficiently long to evaluate such productivity metrics. More bottom-up approaches exploring which outcomes of interdisciplinary research matter to scholars should be considered to verify whether perceived intellectual contribution and perceived commonality are pertinent for researchers involved in interdisciplinary collaborations and whether they lead to long-term productivity outcomes. It is also possible that the period between W1 and W2 was too short for the new collaborations to develop closely so future designs should consider multiple waves spanning across multiple years. Such design would allow to answer the question of whether intellectual appreciation of other disciplines is related to higher productivity with those disciplines.

### Implications and conclusion

The findings of the present research suggest a number of implications for policy-makers and grant funders. Interdisciplinary contact is generally related to higher intellectual appreciation of other disciplines. Increasing opportunities for contact, whether it is through interdisciplinary grants that aim to connect disciplines that are of seemingly different approaches or whether through increasing spaces where such discussions can be happening, policymakers have a clear role in shifting norms and desires to conduct interdisciplinary research. This is particularly important in reducing asymmetries in the ways hard and social sciences perceive one another. If interdisciplinary research is a solution to addressing global issues, then scientists must recognise the ability of other disciplines to contribute intellectually to important research questions.
